# Regulation of Serum Exosomal MicroRNAs in Mice Infected with *Orientia tsutsugamushi*

**DOI:** 10.3390/microorganisms9010080

**Published:** 2020-12-31

**Authors:** Le Jiang, Tatyana Belinskaya, Zhiwen Zhang, Teik-Chye Chan, Wei-Mei Ching, Chien-Chung Chao

**Affiliations:** 1 Viral and Rickettsial Diseases Department, Infectious Diseases Directorate, Naval Medical Research Center, Silver Spring, MD 20910, USA; le.jiang3.ctr@mail.mil (L.J.); tatyana.belinskaya.ctr@mail.mil (T.B.); zhiwen.zhang.ctr@mail.mil (Z.Z.); Teikchye.chan.ctr@mail.mil (T.-C.C.); wei-mei.m.ching.civ@mail.mil (W.-M.C.); 2 Department of Preventive Medicine and Biostatistics, Uniformed Services University of the Health Sciences, Bethesda, MD 20814, USA

**Keywords:** exosome, miRNAs, *O. tsutsugamushi*, scrub typhus, let-7, immune responses

## Abstract

Exosomes are small extracellular vesicles that carry proteins, lipids, and nucleic acids. They are circulated in many body fluids and play an important role in intercellular communications. MicroRNAs (miRNAs), as major components of exosomes, are often regulated in many diseases including bacterial and viral infections. Functionally, exosome-carried miRNAs interact with various immune cells and affect their behavior. Little is known whether exosomal miRNAs are regulated during scrub typhus, a potentially lethal infection caused by intracellular bacteria, *Orientia* *tsutsugamushi*. In the present study, we utilized a scrub typhus mouse model and collected serum at various time points post infection. A custom quantitative PCR array covering 92 murine miRNAs was used to profile serum exosomal miRNAs. A total of 12 miRNAs were found to be significantly up- or down-regulated at least at one time point post infection when compared to uninfected animals. Further analysis identified multiple miRNAs in the let-7 family that were consistently down-regulated at early and late phase of infection. Functionally, serum exosomes isolated from infected mice displayed strong proinflammatory effect when incubated with bone marrow-derived macrophages. Our data revealed dynamic regulations of serum exosomal miRNA during scrub typhus infection, which could significantly influence host immune responses and disease outcome.

## 1. Introduction

Scrub typhus is an acute febrile infectious disease caused by intracellular bacteria of *Orientia tsutsugamushi* that is transmitted by larval feeding of chigger mites (Trombiculidae), mostly *Leptotrombidium* spp. The disease is potentially lethal and endemic in large Asia-Pacific region with over one million cases annually [1]. New pathogenic *Orientia* species have been recently identified and patient cases are expending globally into regions including South America, the Middle East, and Africa [2]. Effective prophylaxis have been long sought after but not available largely due to antigenic heterogeneity of numerous *O. tsutsugamushi* strains, often times circulating in the same geographic area [3]. Initial target cells of this intracellular bacteria have been identified as dendritic cells and monocytes as observed in human eschar tissues [4]. Once infected, *O. tsutsugamushi* is able to evade destruction by macrophages [5] and also subvert dendritic cell functions [6], possibly leading to delayed and constrained adaptive immune responses that are essential for host immune protection against scrub typhus [7].

Exosomes are small extracellular vesicles (usually 40–150 nm) secreted by virtually all cell types. They play important roles in intercellular communication through proteins, lipids and nucleic acids that are specifically packaged within their parental cells of origin [8]. For example, transferring of miRNAs in exosomes derived from regulatory T cells have been shown to suppress pathogenic T cells and systemic inflammation [9]. Exosomes derived from antigen presenting cells (APCs), such as dendritic cells (DCs), can interact with the adaptive immune system and stimulate both T cell and B cell responses [10]. Accumulating evidence has established an important role of exosomes in viral and bacterial infections that could have opposite effect of either pro- or anti-infection, mostly through modulating various layers of host immune responses [11]. Potential regulation and functions of exosomes during *O. tsutsugamushi* infection remains elusive. In the present study, we used a scrub typhus mouse model and investigated miRNA regulations and immunomodulatory functions of serum exosomes isolated from *O. tsutsugamushi*-infected mice.

## 2. Materials and Methods

### 2.1. Mouse Inoculation and Sample Collection

Procedures involving live bacteria of *O. tsutsugamushi* were performed in biosafety level 3 (BSL-3) laboratories. Inoculum (Karp strain) used in this study was produced in CD-1 mice through peritoneal injection of previously purified *O. tsutsugamushi* [12]. Liver and spleen tissue of moribund mice were homogenized, aliquoted, and stored in a −80 °C freezer until use. The inoculum was serially diluted to calculate mLD^50^ [13]. CD-1 mice were challenged via peritoneal injection of 500 × mLD^50^ Karp strain inoculum and blood samples were collected from mice being euthanized at various time points (4 h, 1 day, 2 days, 4 days, 7 days, 10 days and 14 days post infection, *n* = 10 per time point) through cardiac puncture. Blood samples was allowed to clot at room temperature for one hour before being centrifuged at 1000× *g* for 10 min. Sera were carefully removed from clotted blood and stored at −80 °C. In addition, serum samples were collected from uninfected CD-1 mice as controls. All animal procedures were conducted under Walter Reed Army Institute of Research (WRAIR)/Naval Medical Research Center (NMRC) approved IACUC protocols in compliance with the Animal Welfare Act and in accordance with the principles set forth in the “Guide for the Care and Use of Laboratory Animals,” Institute of Laboratory Animals Resources, National Research Council, National Academy Press, 2011.

### 2.2. Determination of Bacteremia and Antibody Titer

Blood clots were first homogenized using 3.5 mm stainless steel beads in Bullet Blender (Next Advance) and DNA was extracted using blood/tissue DNA kit (Qiagen, Hilden, Germany) following manufacturer’s instructions. Quantiative PCR targeting 47 kDa (single copy) and traD (multiple copy) genes of *O. tsutsugamushi* were performed on a 7500 Real-Time PCR System (Applied Biosystems, Foster City, CA, USA) [14,15]. The presence of bacteremia was detectable on day 4 post infection only on traD amplification, but not when using primers for 47 kDa. To determine antibody titers, an enzyme-linked immunosorbent assay (ELISA) was used to detect IgG specific to *O. tsutsugamushi* 56 kDa recombinant protein using serially-diluted serum samples [12]. Starting dilution of the serum was at 1:100 followed by seven 4-fold dilutions (final dilution at 1:1,638,400).

### 2.3. Isolation of Exosomes

Control and serum samples collected at each time point post-infection were pooled from 3–5 mice to a total of 500 µL volume. Differential ultracentrifugation was performed to isolate serum exosomes [16]. Pooled serum was first mixed with 500 µL ice-cold 1X PBS containing EV-Save (FUJIFILM Wako Chemicals, Osaka, Japan) and centrifuged at 1500× *g* for 15 min to clear any cell debris. A subsequent centrifugation at 12,000× *g* for 30 min was performed to precipitate larger extracellular vesicles including macrovesicles and apoptotic bodies. Ice-cold 1X PBS (9.5 mL containing EV-Save) was added to the supernatant and finally, exosomes were precipitated by ultracentrifugation at 120,000× *g* for 2 h. Exosomes were finally resuspended in 100 µL 1X PBS and either used fresh or stored at −80 °C in the freezer until use.

### 2.4. Characterization of Exosomes by Electron Microscopy and Western Blot

Exosomes in 1X PBS were negatively stained with uranyl acetate and examined with a JEM-1400 transmission electron microscope (JEOL, Tokyo, Japan). Images were captured at a magnification of 30,000X with diameter measurements of each exosome. Twenty µL of exosomes were lysed in RIPA buffer (10 mM Tris-Cl, pH 8.0, 150 mM NaCl, 1% Triton X-100, 1% Na-deoxycholate, 1 mM EDTA and 0.05% SDS) and the protein lysate were separated on 4–20% gradient polyacrylamide gels under both reducing (for Calnexin) and non-reducing conditions (for CD9 and CD63). Proteins were then transferred to nitrocellulose membranes, blocked at room temperature for 1 h in 5% milk in 1x TBST buffer and incubated overnight at 4 °C with primary antibodies against Calnexin (C5C9, Cell Signaling Technology), CD9 (MZ3, BioLegend, San Diego, CA, USA), CD63 (NVG-2, BioLegend), and *Orientia* 56 kDa protein (homemade rabbit polyclonal). The membranes were washed 3 times in 1x TBST before incubation with secondary antibodies for 1 h at room temperature. After 3 washes in 1x TBST, ECL substrate (ThermoFisher, Waltham, MA, USA) was added to the membranes. Western blot signals with various exposure times were then captured in a ChemiDoc XRS+ system (Bio-Rad, Hercules, CA, USA).

### 2.5. Nanoparticle Tracking Analysis

Exosomes were diluted 80 times with 1X PBS and loaded into low volume flow cell assembly of NanoSight NS300 (Malvern Panalytical, Malvern, the United Kingdom) equipped with blue laser (488 nm) and a scientific CMOS camera. Ten videos (60 s each, 25 frames per second) were captured for serum exosomes at each time point and analyzed using NanoSight NTA 3.2 software. Camera level was set at 13 and the detection threshold was set at 10.

### 2.6. MicroRNA Profiling

Sixty µL of exosomes were used for miRNA extraction using miRNeasy mini kit (Qiagen, Hilden, Germany). Prior to extraction, UniSp6 RNA was added to each sample as the spike-in control. To profile miRNA expression, a custom miRCURY LNA miRNA PCR panel (Qiagen, Hilden, Germany) was designed for 92 miRNAs that are abundantly-expressed in mouse serum [17]. Identities and target sequences of these miRNAs are listed in Supplementary Table S1. Universal reverse transcription was performed using miRCURY LNA RT kit (Qiagen, Hilden, Germany). Quantification of miRNAs were carried out on a 7500 Real-Time PCR System using miRCURY LNA SYBR green PCR kit (Qiagen, Hilden, Germany) following manufacturer’s instructions. Threshold for all PCR plates was set at 0.2 (Supplementary Figure S1). Raw C_T_ (cycle threshold) value from all samples were first adjusted using UniSp3 inter plate calibrator (IPC). Twelve miRNAs with average C_T_ values exceeding 35 at any time point were removed from further analysis. C_T_ values from 80 miRNAs were normalized using array mean method [18] and expression levels of individual miRNA were normalized to the mean expression level of all miRNAs. NormFinder software [19] was used to rank expression stability and identified two endogenous normalizer miRNAs (mmu-miR-27a-3p and mmu-miR-30d-5p), the average of which was used to normalize miRNA expression levels in assays quantifying the limited number of miRNAs.

### 2.7. Treatment of Bone-Marrow Derived Macrophages and mRNA Quantification

Bone marrow-derived macrophages were derived as previously described [20]. Bone marrow cells were isolated from normal CD-1 mice and cultured with 20 ng/mL recombinant Macrophage Colony-Stimulating Factor (M-CSF, PROSPEC) for 6 days. Macrophages were washed twice with 1X PBS and replaced with media containing exosome-depleted FBS (ThermoFisher, Waltham, MA, USA). The next day, 2 × 10^5^ macrophages were seeded into each well of a 24-well tissue culture plate. After cell attachment, 70 µL (approximately 1 × 10^9^ particles) of exosomes were added to each well. Twenty-four hours later, macrophages were harvested, and total RNA was extracted using miRNeasy mini kit (Qiagen, Hilden, Germany). Reverse transcription was carried out using 500 ng of total RNA RT^2^ first strand kit for cDNA synthesis (Qiagen, Hilden, Germany) with the step of genomic DNA elimination. Quantitative PCR was conducted on a 7500 Real-Time PCR System using standard protocol and the following primers for murine genes: *Ccl5*, forward, TGCAGAGGACTCTGAGACAGC, reverse, GAGTGGTGTCCGAGCCATA; *Il1b*, forward, AGTTGACGGACCCCAAAAG, reverse, AGCTGGATGCTCTCATCAGG; *Tgfb1*, forward, TGGAGCAACATGTGGAACTC, reverse, GTCAGCAGCCGGTTACCA; *Il6*, forward, GCTACCAAACTGGATATAATCAGGA, reverse, CCAGGTAGCTATGGTACTCCAGAA; *Il10*, forward, CAGAGCCACATGCTCCTAGA, reverse, TGTCCAGCTGGTCCTTTGTT; *Tnf*, forward, TCTTCTCATTCCTGCTTGTGG, reverse, GGTCTGGGCCATAGAACTGA and endogenous housekeeping control *Gapdh*, forward, GGGTTCCTATAAATACGGACTGC, reverse, CCATTTTGTCTACGGGACGA. Relative expression levels of these genes normalized to *Gapdh* were calculated based on the C_T_ values [21].

### 2.8. Statistical Analysis

Multiple t tests in GraphPad Prism (version 8.3.1) were used to compare miRNA expression levels between control and various time points post infection. One-way ANOVA followed by Tukey’s multiple comparisons test was used to compare mRNA expression levels in macrophages among various treatment groups. A p-value equal to or less than 0.05 was considered statistically significant.

## 3. Results

### 3.1. Characterization of Serum Exosomes Isolated from Orientia Tsutsugamushi-Infected Mice

**Figure 1:**
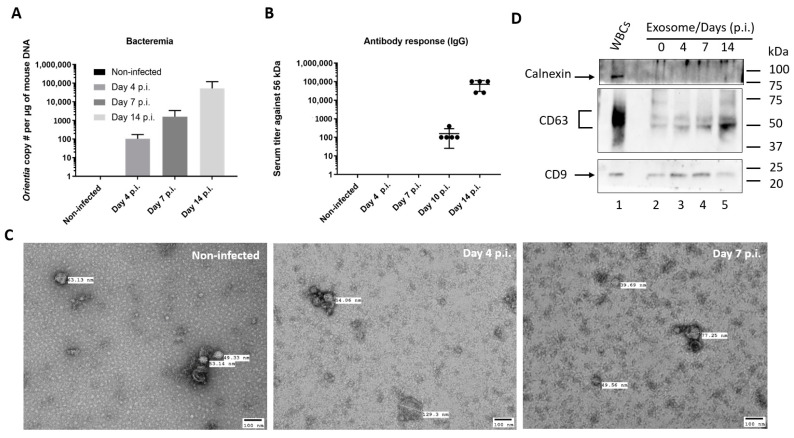
Serum exosomes isolated from *O. tsutsugamushi*-infected CD-1 mice. (**A**) Blood DNA was extracted from non-infected and infected mice (*n* = 3 per group) and amount of *Orientia* was determined by quantitative PCR. The experiment was repeated twice and representative data were shown. Error bar, standard deviation. (**B**) IgG antibody titers against *Orientia* major surface antigen, 56 kDa protein, was determined by ELISA using four-fold serially-diluted mouse serum (*n* = 5 per time point). Error bar, standard deviation. (**C**) Representative electron microscope images of exosomes isolated from serum of non-infected mice or *Orientia*-infected mice 4 days and 7 days after infection. (**D**) Exosomes and white blood cells (WBCs) were lysed and analyzed by Western blot for the presence of Calnexin, CD9 and CD63 proteins. Lane 1, WBC lysate from CD-1 mice; Lane 2-5, lysate of 20 µL serum exosomes from non-infected or infected mice of day 4, 7 and 14 post infection (p.i.).

To study serum exosomes during *O. tsutsugamushi* infection, a lethal murine model of scrub typhus was used as previously conducted in our laboratory [12]. Outbred CD-1 mice were challenged through injection of 500x mLD_50_ inoculum (Karp strain) into peritoneal cavity. Infected mice became sick around day 10 and 40% of mice died on day 14. All mice succumbed to infection by day 21 post infection. Presence of *O. tsutsugamushi* was detected on day 4 with very low level of bacteremia, which increased exponentially as infection proceeded to day 7 and day 14 (Figure 1A), corresponding to the appearance of morbidity such as ruffled fur on day 7 and beginning of lethality on day 14. Antibodies against immunodominant 56 kDa protein [22] became detectable on day 10 and serum titers exceeded 25,600 in all mice on day 14 post infection (Figure 1B). These observations demonstrated proper inoculation followed by anticipated disease progression and humoral immune responses in the infected mice. Subsequently, exosomes were isolated using pooled sera from either control or infected mice using differential ultracentrifugation and examined under electron microscope. Exosomes appeared as spheres with expected sizes between 40–130 nm (Figure 1C). Further analysis using Nanoparticle Tracking Analysis (NTA) revealed that vast majority of the purified exosomes were below 200 nm and average modal size ranged from 99 to 128 nm (Supplementary Figure S2A–D). In addition, particle concentrations were determined to vary between 1.3 to 2.1 × 10^10^ /mL and there was no significant difference among control and infected groups. To further characterize these exosomes, Western blots were performed, and recognized protein markers specifically enriched in exosomes. Tetraspanins of CD9 and CD63 were readily detectable in serum exosomes isolated at various time points post infection (Figure 1D). Calnexin, an integral protein of endoplasmic reticulum, was present in white blood cells, but not present in the serum exosomes, indicating absence of host cell or protein contamination. These data demonstrated successful isolation of serum exosomes from mice infected with *O. tsutsugamushi*.

### 3.2. Profile of Serum Exosomal microRNAs

**Figure 2:**
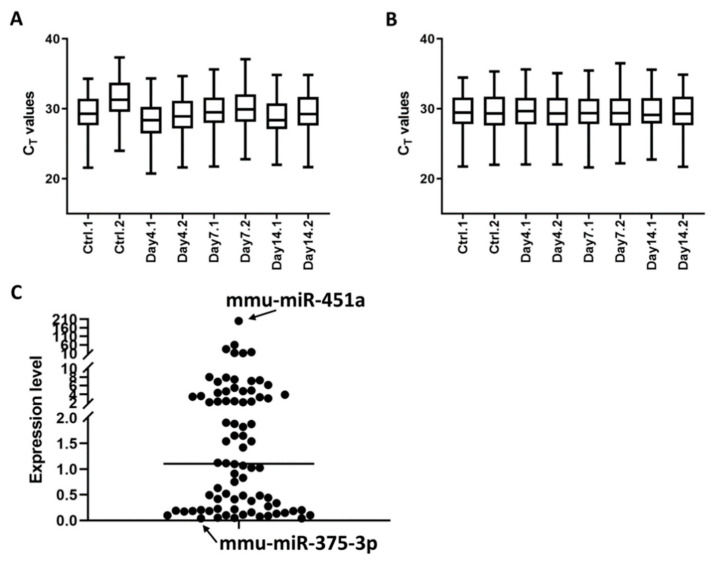
MicroRNA expression profiles of serum exosomes from non-infected and *Orientia*-infected mice. (**A**) Box plots depicting C_T_ values of 80 miRNAs determined through a custom miRCURY LNA miRNA quantitative PCR array. For each time point, data were generated from two Scheme 3. mice. (**B**) C_T_ values were normalized by mean expression value of all 80 expressed miRNAs and box plots after normalization were shown. (**C**) Relative expression levels of 80 individual miRNA in serum exosomes from non-infected mice after normalization to the array mean expression. Arrow heads point to the two miRNAs with the highest and lowest expression levels. Vertical line denotes median expression level.

MiRNAs are major component of exosomes and can functionally modulate immune responses during pathogen invasion [23,24]. We set out to investigate serum exosomal miRNA expression profile and utilized a custom miRCURY LNA miRNA PCR array that targets 92 miRNAs (Supplementary Table S1) from the top 100 abundantly-expressed serum exosomal miRNAs recently identified through RNAseq [17]. MiRNAs purified from exosomes isolated at various time points were reverse transcribed and amplified on the PCR array. In line with the RNAseq report, majority of the 92 miRNAs are detectable on the PCR array (Supplementary Figure S1 and Table S1). Among these, 80 miRNAs were found to be consistently expressed (average C_T_ value ≤ 35) in serum exosomes isolated at 0, 4, 7, and 14 d post infection and were further analyzed. To reduce variations that might occur during exosome isolation, RNA extraction and reverse transcription, a method of normalization using the mean expression value of all expressed miRNAs [18] was applied (Figure 2A,B). As a result, average coefficient of variation (CV) on C_T_ values decreased from 3.78% to 1.87%. Relative expression levels of the 80 miRNAs in the non-infected serum varied widely from 0.04 (mmu-miR-375-3p) to 201.03 (mmu-miR-451a), approximately 5000-fold difference in terms of abundance (Figure 2C). The identification of stably expressed miRNAs is essential for the relative quantification of a limited number of miRNAs. NormFinder [19] ranked mmu-miR-27a-3p and mmu-miR-30d-5p as the two most stable miRNAs in serum exosomes (Supplementary Figure S3), which were later used as normalizers for expression quantification of the let-7 miRNAs.

### 3.3. Serum Exosomal microRNAs Are Regulated during Orientia Tsutsugamushi Infection

**Figure 3:**
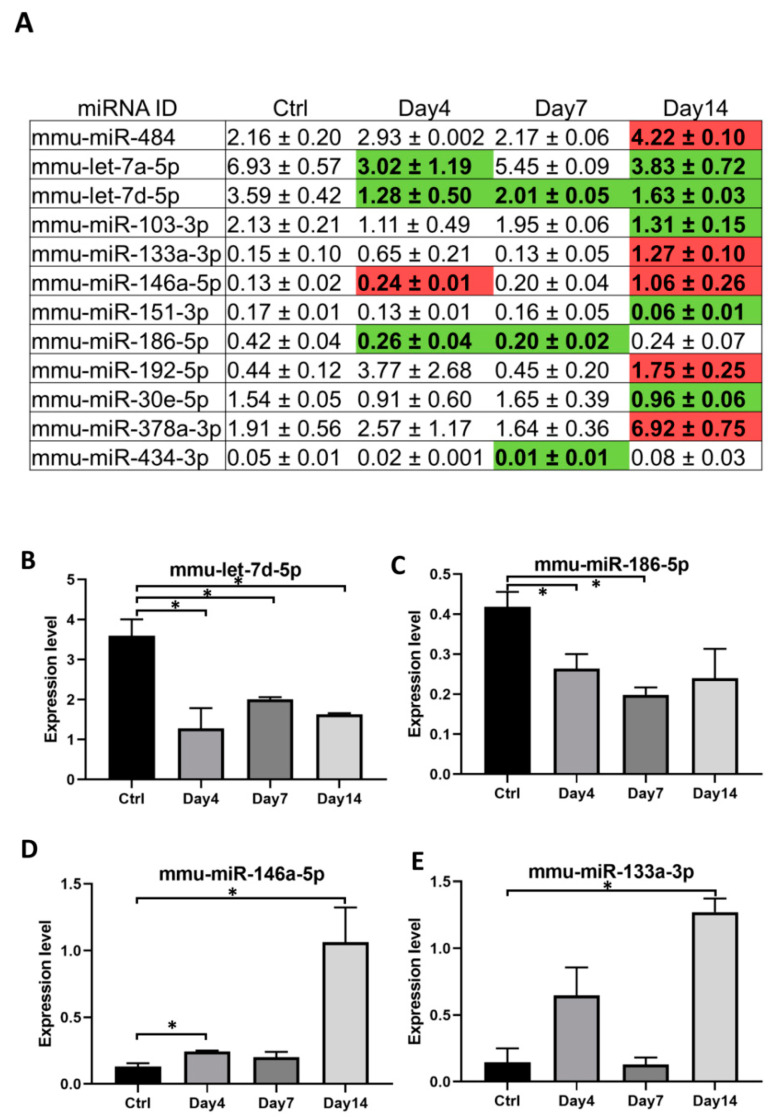
Abundance of serum exosomal microRNAs are regulated during *O. tsutsugamushi* infection. (**A**) Expression levels of thirteen miRNAs were found to be significantly regulated post infection when compared to the controls with more than 1.5 fold changes (mean ± standard deviation). Red boxes, upregulation; green boxes, downregulation. B to E, Examples of four exosomal miRNAs that were either repressed (**B**,**C**) or activated (**D**,**E**) during *Orientia* infection. Error bar, standard deviation from two pooled biological replicates. * *p*-value ≤ 0.05.

Expression of specific subset of miRNAs can be regulated upon pathogen invasion [25]. To identify any exosomal miRNAs significantly modulated during the course of *O. tsutsugamushi* infection, we compared expression levels of each of the 80 miRNA at 4, 7, and 14 d post infection to those of the non-infected control. A total of 12 miRNAs were found to be regulated significantly with fold changes ≥1.5 at least at one time point post infection (Figure 3A). These miRNAs were either repressed (5 miRNAs, Figure 3B,C) or activated (7 miRNAs, Figure 3D,E) due to *Orientia* infection. Potential functions of these 12 miRNAs as a group were analyzed by mirPath v.3 [26] and the top three significant pathways that could be modulated by these miRNAs included extracellular matrix (ECM)–receptor interaction, prion disease, and MAPK signaling pathway.

### 3.4. Members of Let-7 Family of microRNAs Are Dynamically Regulated during Orientia Tsutsugamushi Infection

**Figure 4:**
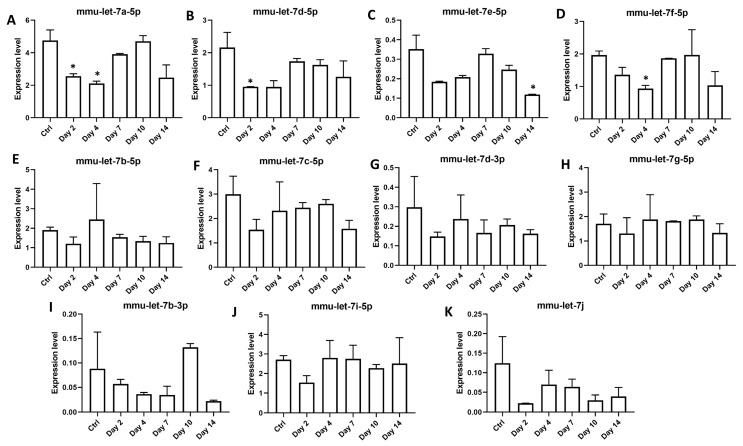
Members of let-7 family of microRNAs are dynamically regulated during infection. Expression levels of eleven members of the let-7 family (panel **A**–**K**, normalized to the average expression of mmu-miR-27a-3p and mmu-miR-30d-5p) in exosomes isolated from serum of control or infected mice (2, 4, 7, 10, 14 days post infection). Exosomes were isolated from two pools of sera for each time point. Error bar, standard deviation from two pooled biological replicates. The experiment was repeated twice and representative data were shown. * *p*-value ≤ 0.05 comparing with the controls.

Interestingly, two miRNAs from the let-7 family were consistently repressed during *O. tsutsugamushi* infection (Figure 3A,B). Members of let-7 miRNAs have been shown to impact host immune responses through regulation on toll like receptors, cytokine release or other immune pathways [27,28,29]. To see whether other members of this family were also regulated during *Orientia* infection, we expanded our expression analysis to include other known murine let-7 miRNAs [30] and included additional time points of day 2 and day 10 post infection (Supplementary Table S2). Eleven miRNAs of this family were consistently expressed in serum exosomes during *Orientia* infection and four members of let-7 miRNAs, including let-7 a,d,f cluster [31] and let-7e-5p, were found to be significantly repressed during early (2–4 days) and late (14 days) phases of *Orientia* infection (Figure 4A–D). Interestingly, this repression was completed relieved on day 7 and day 10, suggestive of a very dynamic regulation on this set of let-7 miRNAs.

### 3.5. Serum Exosomes from Orientia Tsutsugamushi-Infected Mice Are Proinflammatory

**Figure 5:**
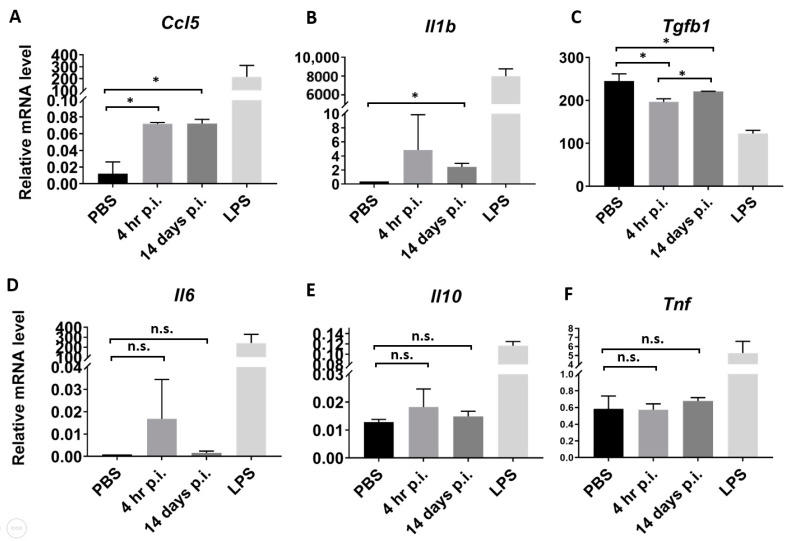
Serum exosomes from *O. tsutsugamushi*-infected mice modulate cytokine expression in macrophages. BMDMs were treated with PBS (vehicle control), serum exosomes from infected mice or LPS (5 µg/mL) for 24 h and mRNA transcript levels of cytokines or chemokines (**A**, *Ccl5*; **B**, *Il1b*; **C**, *Tgfb1*; **D**, *Il6*; **E**, *Il10* and **F**, *Tnf*) were determined and normalized to the expression of *Gapdh* as endogenous control (n.s., not significant; * *p*-value ≤ 0.05). The experiment was repeated twice and representative data were shown. Error bar, standard deviation.

Exosomes have been shown in a number of studies to impact immune responses upon pathogen infections [11]. Given the immunomodulatory let-7 miRNAs being expressed and regulated during *Orientia* infection (Figure 4), we treated BMDM with exosomes isolated from mouse sera (4 h and 14 days post infection) and measured mRNA transcript levels of several key cytokines and chemokines released by macrophages [32]. Lipopolysaccharide (LPS) treatment resulted in dramatic regulations in all targets examined (Figure 5). Transcript level of *Ccl5*, a potent chemoattractant for lymphocytes, increased six-fold after treatment by serum exosomes (Figure 5A). Transcription of *Il1b,* a potent proinflammatory cytokine, also increased more than six-fold in exosome-treated BMDM (Figure 5B). *Il-6* was also induced upon exosome treatment, although not statistically significant. Protein product of *Tgfb1* is an anti-inflammatory cytokine that has inhibitory effects on both T and B lymphocytes. Its transcription was significantly down-regulated upon exosome treatment (Figure 5C). *Tnf* and anti-inflammatory cytokine of *Il-10* genes were not regulated. Overall, these data indicated strong proinflammatory function of exosomes derived from *Orientia*-infected mouse serum.

## 4. Discussion

To our knowledge, the present study represents the first attempt to profile exosomal miRNAs and to explore immunological functions of serum exosomes during the course of *O. tsutsugamushi* infection. Using a custom PCR array covering 92 murine miRNAs, 12 miRNAs were found to be significantly regulated by more than 1.5 folds at least at one time point post infection. There were more miRNAs being modulated during late (10 miRNAs on day 14) phase of infection, compared to day 4 or day 7 where only four and three miRNAs were modulated respectively (Figure 3A). The combinational effects of these miRNA regulations at each time point, particularly towards transcriptional modulation, warrant further investigations. While the immunomodulatory function of exosomes as demonstrated in Figure 5 might be attributed by miRNAs, critical involvement of proteins and other components of exosomes could not be ruled out. Nevertheless, the proinflammatory effect of exosomes (upregulation of *Ccl5, Il-1b,* and downregulation of *Tgfb1*) probably will contribute to the activation of host innate and adaptive immune responses against *O. tsutsugamushi.* Besides involvement of miRNAs and host proteins, exosomes secreted from pathogen-infected cells have been shown to carry specific antigens that possess immunomodulatory functions [33]. In addition, *Orientia*, like many other Gram-negative bacteria, has been reported to secrete outer membrane vesicles (OMVs) carrying 56 kDa antigen [34]. Since OMVs are similar in size as exosomes, they are likely to be isolated together with serum exosomes, especially when bacteremia level increases dramatically at later phase of infection (Figure 1A). However, we failed to detect any 56 kDa antigen in exosomes isolated from serum on day 7 and 14 post infection (Supplementary Figure S4), suggesting that *Orientia* major antigens might not have significant contributions to the immunomodulatory functions of exosomes observed in our study.

It is intriguing whether similar exosomal miRNA regulations and proinflammatory functions of exosomes occur in human scrub typhus cases. Some of the miRNA regulations were evident as early as day 2 post infection (Figure 4A), even before the appearance of bacteremia on day 4 (Figure 1A), suggesting their potential value as biomarkers for early diagnosis of scrub typhus. While both serum and plasma have been extensively used to study exosomal miRNAs and to identify potential biomarkers [35], compositions of extracellular vesicles in serum might be affected during blood coagulation due to involvement of platelets [36,37]. As serum is investigated in the present study, it remains to be determined whether exosomes isolated from platelet-free plasma will share similar miRNA regulations and functional properties.

Let-7 family of miRNAs are highly conserved across species [38] and their roles in modulating immune responses have been recently established in serval infectious diseases [39]. Down-regulation of let-7 family miRNAs have been reported in both viral [40] and bacterial infections [27,28,29], which in turn alters expression of numerous critical targets in immune pathways. Similarly, we found four members of the let-7 family miRNAs to be repressed in serum exosomes upon *Orientia* infection. Such repressions occurred either early on day2/day 4 or late on day 14 post infection and interestingly, these repressions were somehow relieved on day 7 and day 10 (Figure 4A–D). Mechanism(s) that lead to the dynamic abundance of specific miRNAs during *Orientia* infection remain to be investigated. These dynamic changes in the exosomal miRNA composition, and potentially in other exosomal components as well, might have differentially affected immune cell behavior, such as levels of *Tgfb1* expression in macrophages during the early (4 h) vs. late (14 days) phase of infection (Figure 5C). Interestingly, three of these regulated miRNAs (let-7a, 7d, and 7f) reside in a cluster within a ~2 Kb region on murine chromosome 13. Genetic manipulation of this cluster demonstrated its critical role in induction of IL-6 cytokine in macrophages [31]. IL-6 is a pleiotropic cytokine that regulate a range of downstream targets, resulting in activation and regulation of immune responses. In line with this, we observed increased *Il-6* transcription by almost 20-fold after treatment using serum exosomes 4 h post infection (although this did not reach statistical significance due to large variations). Besides members of the let-7 miRNAs, dynamic regulations are also evident on a number of other serum exosomal miRNAs during *Orientia* infection (Figure 3) and these miRNAs may act in concert resulting in temporal modulations of many pathways recently discovered using a similar mouse model in our laboratory [41]. More comprehensive integrative studies of mRNA/miRNA profiles, innate/adaptive immune responses, and pathogenies during various stages of *Orientia* infection may provide a better understanding of host responses and potential intervention points to control scrub typhus.

## Data Availability

Data are contained within the article or supplementary materials.

## Supplementary Materials

The following are available online at https://www.mdpi.com/2076-2607/9/1/80/s1. Figure S1. Representative image of an amplification plot of the custom miRCURY LNA miRNA PCR array covering 92 miRNAs; Figure S2. Particle size distribution and concentrations of serum exosomes isolated from control and infected mice; Figure S3. C_T_ values of the most stably-expressed miRNAs (A, mmu-miR-27a-3p and B, mmu-miR-30d-5p) in serum exosome during *Orientia* infection; Figure S4. Absence of 56 kDa antigen in serum exosomes isolated from mice on day 7 and 14 post infection; Table S1. Raw C_T_ values of 92 murine miRNAs amplified on a custom miRCURY LNA miRNA PCR array; Table S2. Raw C_T_ values of let-7 family miRNAs and controls amplified using miRCURY LNA assays.
