# Supplementary figures and images for: Regulation of Serum Exosomal MicroRNAs in Mice Infected with Orientia tsutsugamushi
Source: Microorganisms. 2020 Dec 31;9(1):80. doi: 10.3390/microorganisms9010080 (PMC7823836; doi:10.3390/microorganisms9010080)

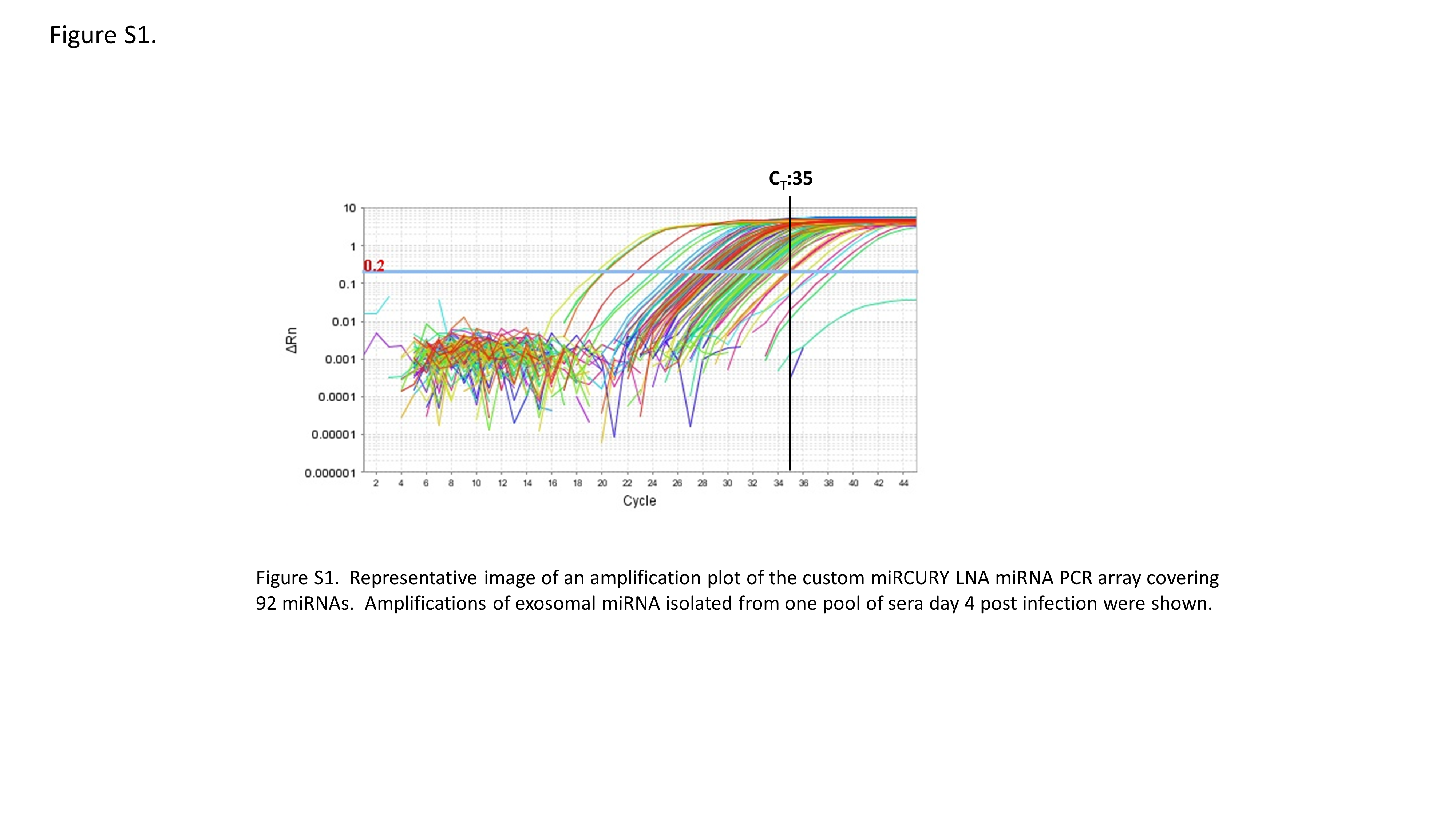

Supplement: Supplementary file 1 [file microorganisms-09-00080-s001.zip › Supplementary files/Figure S1.TIF]

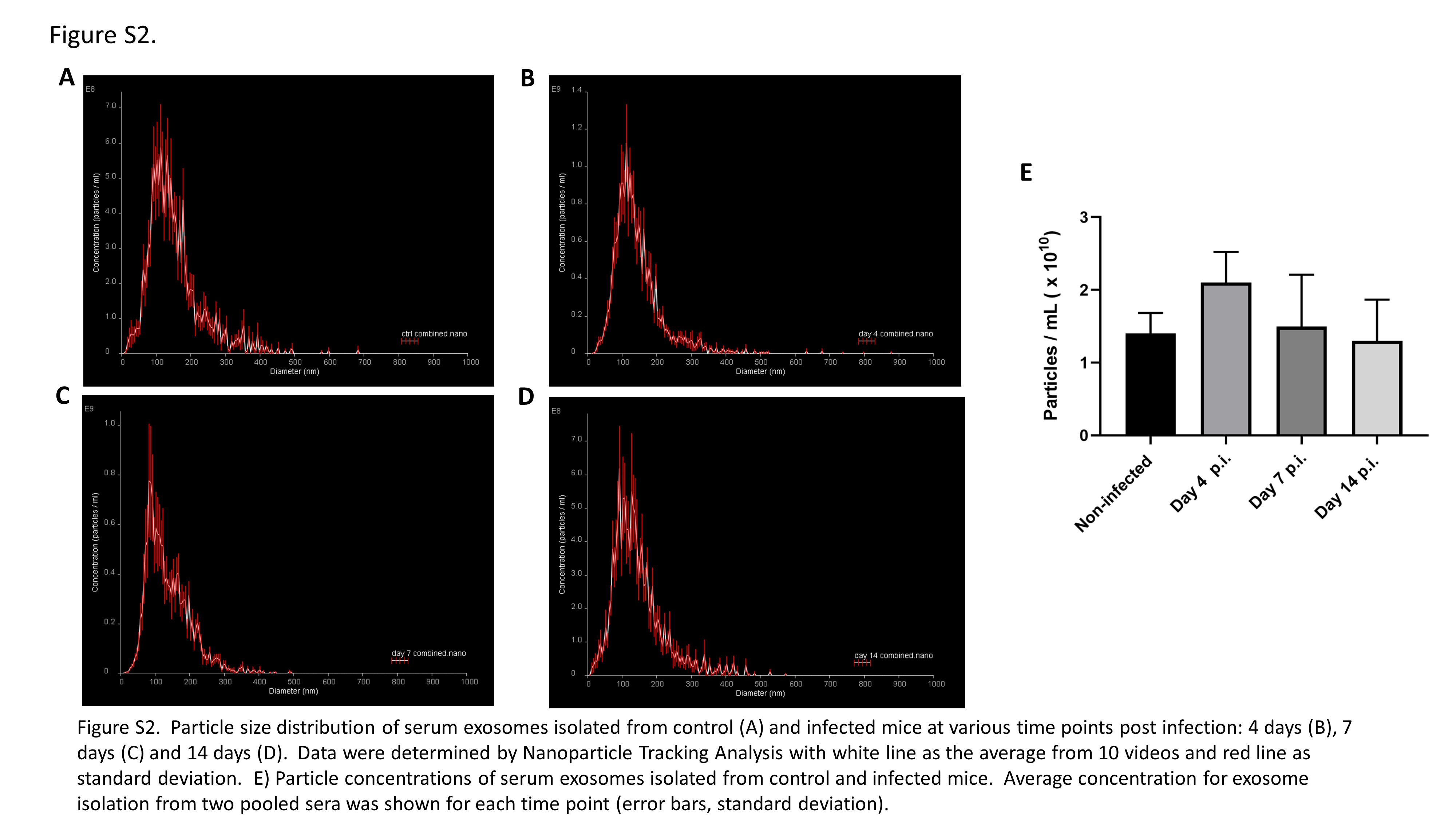

Supplement: Supplementary file 1 [file microorganisms-09-00080-s001.zip › Supplementary files/Figure S2.TIF]

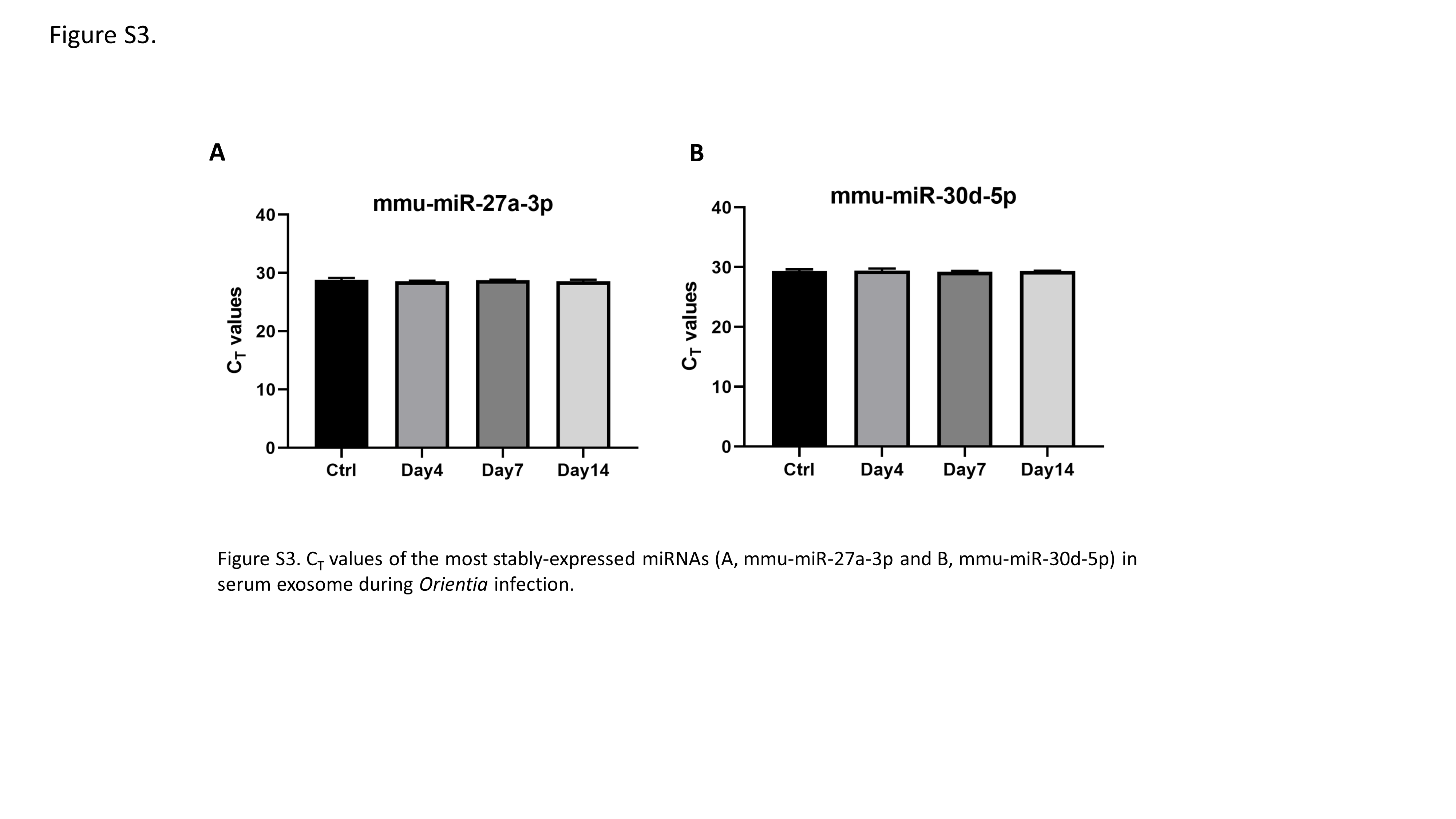

Supplement: Supplementary file 1 [file microorganisms-09-00080-s001.zip › Supplementary files/Figure S3.TIF]

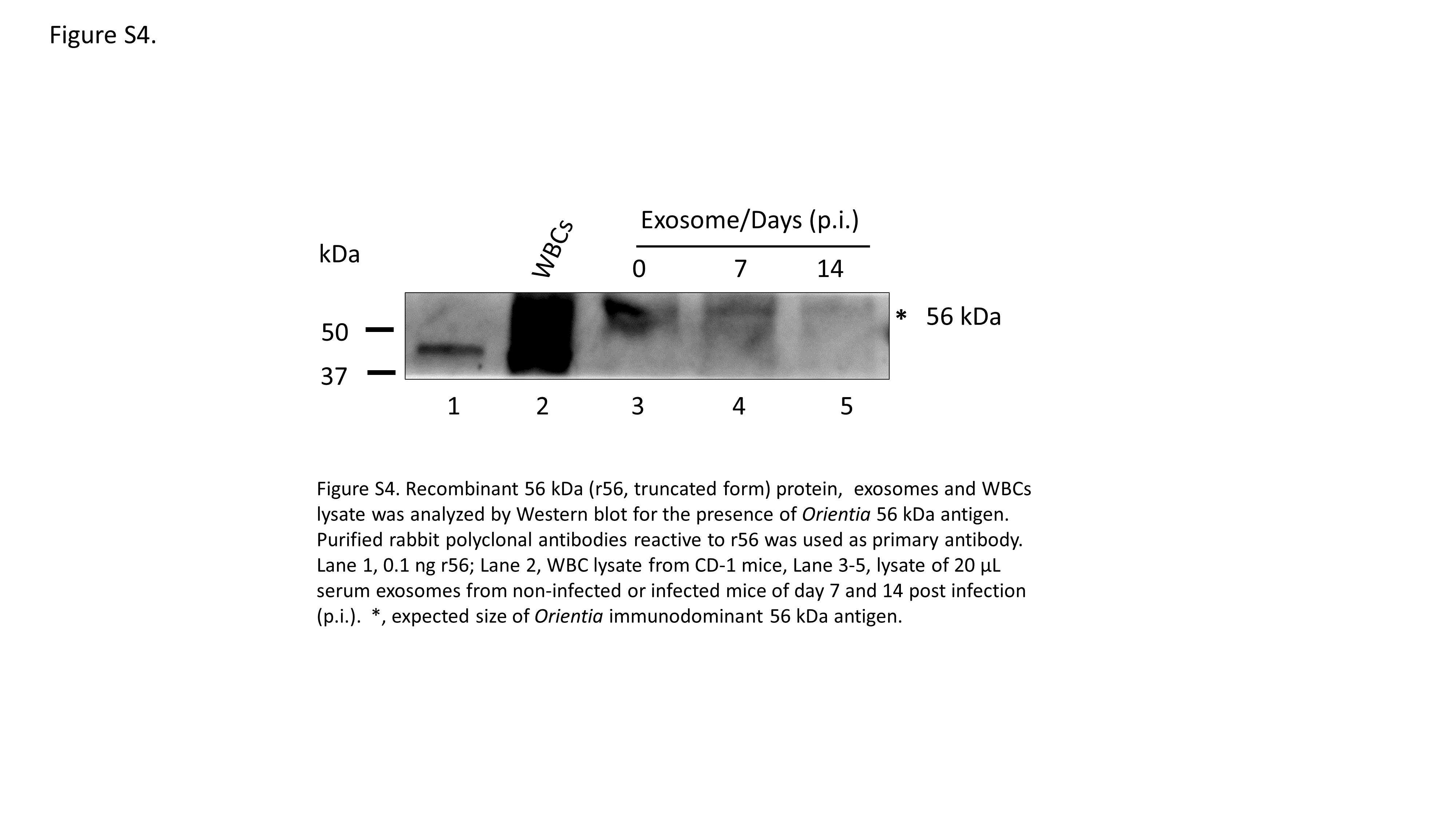

Supplement: Supplementary file 1 [file microorganisms-09-00080-s001.zip › Supplementary files/Figure S4.TIF]
